# Iterative development of MobileMums: a physical activity intervention for women with young children

**DOI:** 10.1186/1479-5868-9-151

**Published:** 2012-12-20

**Authors:** Brianna S Fjeldsoe, Yvette D Miller, Jasmine L O’Brien, Alison L Marshall

**Affiliations:** 1 School of Population Health, Cancer Prevention Research Centre, The University of Queensland, Herston, Queensland, Australia; 2 School of Psychology, Queensland Centre for Mothers and Babies, The University of Queensland, St Lucia, Queensland, Australia; 3 School of Public Health, Institute of Health and Biomedical Innovation, Queensland University of Technology, Kelvin Grove, Queensland, Australia

**Keywords:** Mobile phone, Exercise, Postnatal, mHealth, Text messaging, SMS

## Abstract

**Background:**

To describe the iterative development process and final version of ‘MobileMums’: a physical activity intervention for women with young children (<5 years) delivered primarily via mobile telephone (mHealth) short messaging service (SMS).

**Methods:**

MobileMums development followed the five steps outlined in the mHealth development and evaluation framework: 1) conceptualization (critique of literature and theory); 2) formative research (focus groups, n= 48); 3) pre-testing (qualitative pilot of intervention components, n= 12); 4) pilot testing (pilot RCT, n= 88); and, 5) qualitative evaluation of the refined intervention (n= 6).

**Results:**

Key findings identified throughout the development process that shaped the MobileMums program were the need for: behaviour change techniques to be grounded in Social Cognitive Theory; tailored SMS content; two-way SMS interaction; rapport between SMS sender and recipient; an automated software platform to generate and send SMS; and, flexibility in location of a face-to-face delivered component.

**Conclusions:**

The final version of MobileMums is flexible and adaptive to individual participant’s physical activity goals, expectations and environment. MobileMums is being evaluated in a community-based randomised controlled efficacy trial (ACTRN12611000481976).

## Background

Evidence-based advancements in health behaviour change research require thorough and transparent reporting of intervention development and content. Recent literature has called for: 1) interventions to be developed based on behaviour change theory, published evidence and formative research [1,2]; and, 2) publication of the intervention development process, including details of the final program content using standardised descriptors and language [3-5]. To date, few authors have reported on their intervention development methods, and published evaluations rarely provide the level of detail required to replicate the intervention. However, there are notable examples of authors who have provided this level of detail in publications e.g., [6-8]. These gaps in the literature limit our ability to advance health behaviour change practice and policy [2].

There are published frameworks that guide the process of developing health behaviour change interventions, such as: Intervention Mapping [9], and the Medical Research Council’s Framework for developing and evaluating complex interventions to improve health [10]. More recently, three frameworks have been specifically created to guide the development of mHealth interventions (interventions primarily delivered via mobile telephone technology): the Multiphase Optimisation Strategy (MOST) [11]; the Sequential Multiple Assignment Randomized Trial (SMART) [11]; and, the mHealth Development and Evaluation framework [12]. A common theme among these frameworks is the integration of information sources to inform intervention design, including published evidence, theory and formative research with the target group. The MOST and SMART frameworks involve multiple phases of randomised trials of separate intervention components to determine the best combination. However, the MOST framework violates the common assumption of many behaviour change theories: that theoretical constructs (such as outcome expectancies, self-regulation etc.) are interconnected and posited to influence behaviour when targeted in concert, not in isolation. Furthermore, the resources and time required to recruit participants and test intervention components in multiple, sufficiently-powered experimental trials can be prohibitive. The SMART framework is also specific to time-varying adaptive interventions (where intervention content is adapted over time depending on participant behaviour change progress), which was never the intention of the intervention described in this paper. The mHealth framework is an iterative process of refining complete mobile phone interventions based on concurrent and sequential quantitative and qualitative research among the target group. This framework maintains a strong focus on dissemination throughout the development process. For these reasons, we have used the mHealth framework to describe the development of our intervention.

The aim of this paper is to describe the iterative development process and final version of a physical activity intervention designed specifically for women with young children (<5 years). The intervention, called ‘MobileMums’, is a 12-week program delivered primarily via mobile telephone short messaging service (SMS). After describing the development process using the mHealth framework, we will describe the intervention components necessary to adequately replicate the MobileMums intervention [4] using the language defined in the taxonomy of behaviour change techniques [3] to demonstrate how the intervention content was guided by Social Cognitive Theory [13,14].

## Methods and results

**Figure 1:**
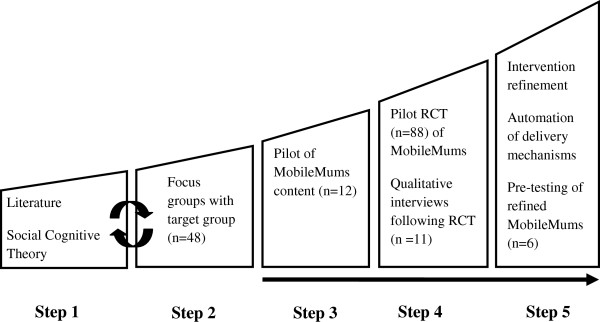
Methods used to inform the development of MobileMums based on the five step mHealth Development and Evaluation framework.

The development of MobileMums followed the five steps of the mHealth framework [12]: 1) conceptualisation; 2) formative research; 3) pre testing; 4) pilot testing; and, 5) qualitative research for intervention refinement. In our application of the framework, Steps 1 and 2 were conducted concurrently; after which three iterations of the MobileMums program were tested sequentially through Steps 3 to 5 (Figure 1). The findings of each step were used to refine the MobileMums program before subjecting it to the next step in the development and evaluation framework: consequently this paper describes the methods and results of each step sequentially.

### Step 1 conceptualisation

MobileMums was conceptualised following a review of existing evidence and theories of behaviour change. Epidemiological evidence clearly identified women with young children as a priority group for physical activity intervention. Women with young children are consistently shown to be less active than women of the same age without children [15,16] or women with older children [15,17,18]. The child-rearing stage of life has also been identified as a time when behaviours and routines are naturally changing [19] and when women’s behaviour can influence that of her immediate family [19].

The review of intervention literature revealed that most physical activity interventions targeting women with young children used community-based pram walking groups [20-23]. Overall, the evidence that this intervention approach assisted women to increase their physical activity was equivocal and most studies reported poor group attendance rates. Greater behaviour change success was observed following theory-based face-to-face (F2F) counselling interventions. However, this intervention approach was resource intensive, and also suffered low participant adherence [24-28]. Collectively, the evidence indicated that women with young children would benefit most from theory-based, personalised interventions that offer flexible delivery mechanisms to increase adherence.

Social Cognitive Theory [13,14] has been widely used in physical activity intervention research. Constructs from this theory have been endorsed as important for eliciting initiation of physical activity behaviour change in the general adult population [29] and specifically among women with young children [25,30]. The underlying principle of this theory is reciprocal determinism: the way an individual’s cognitions, environmental perceptions and behaviour interact and influence each other [13,14]. The balance of influences emphasised in this theory were congruent with the theoretical constructs (i.e., social support, self efficacy) found to elicit behaviour change in the two mediator analyses previously conducted on physical activity interventions among women with young children [25,30]. This evidence led to the apriori selection of Social Cognitive Theory as the theoretical foundation of MobileMums in favour of other possible health behaviour theories (e.g. Theory of Planned Behaviour).

### Step 2 formative research

In order to refine the scope of the intervention content and regimen [12], we conducted focus groups with women with young children to explore their perceptions of and needs for physical activity interventions. Women were recruited via posters and flyers displayed in general practice clinics, Playgroup locations, the public hospital antenatal clinic, and the public library of a community located in Queensland, Australia. To be eligible to participate, women had to have at least one child aged less than 5 years, and be able to speak and understand English. Consistent with standard methods for conducting qualitative research [31], recruitment continued until no new information was collected from each new focus group. A moderator guide was used to standardise discussion topics across groups. Each group was audio recorded and transcribed verbatim. Transcripts were analysed by two independent researchers (BF and AM) and moderated by a third researcher (YM). All analysts were experienced in conducting qualitative research. The thematic analysis followed a systematic and iterative process, whereby major themes and categories were identified and used to classify data from each group, then across the complete dataset [31]. A University Human Research Ethics Committee approved the study.

Forty-eight women with young children (aged between 16 and 45 years) participated in one of eight focus groups. Most participants (41/48) were married and a third (16/48) were pregnant at the time of participating. One third of participants (16/48) had a weekly household income less than AUD $600 per week and one quarter (13/48) had a Year 10 equivalent level education.

Nearly all participants were aware of the Australian physical activity recommendations [32] for duration (i.e., 30-minutes per day), but were less informed about the recommended frequency (i.e., most days of the week). Most participants understood moderate-intensity activities were sufficient for health benefit. Walking and water-based activities were the most common activities identified as being health-enhancing. Participants most commonly reported a desire to do more walking, swimming and gym classes. The availability of free, convenient, trustworthy childcare was revealed as the primary perceived need for women to increase their physical activity. Other perceived facilitators for physical activity included opportunities to avoid heat via indoor activities and opportunities for access to group activities, such as mothers walking groups, to assist with motivation.

*“..the best bit* [about the walking groups] *was just getting to talk to other mums week after week. You developed friendships, and they encouraged you to come back the next week and always had something useful to share”.*

Some participants raised potential barriers to attending organised group sessions such as allocated times not suiting their weekly schedules or their child’s disposition on the day, finding trustworthy childcare and proximity of a group location to their home. Women most preferred to receive informal advice about physical activity from acquaintances (e.g., partner, female friends) that were currently active or had a history of being active.

When discussing flexible modes of receiving support for physical activity, participants in each group raised the use of mobile telephones. On further probing by the moderator it was found that all participants owned a mobile telephone, carried them all the time and primarily used them for SMS. Outgoing calls were limited to ‘emergencies’ due to high call costs and clarity of service. Participants liked the idea of receiving personalised health-related messages via SMS. SMS were preferred over calls because messages could be read or responded to at a time that was personally convenient.

“it’s [SMS] not like a phone call…you are probably doing something like bathing a kid or actually busy or something and you’ve got to get to the phone. With a message you can always get to it later”

Following the findings of the focus groups, we systematically reviewed the available evidence on use of SMS to deliver behaviour change interventions [33]. From this review, we concluded that MobileMums would need to: deliver tailored SMS content to individuals; establish rapport between the SMS recipient and sender; and, encourage two-way SMS communication. Since this review was published in 2009 another five reviews of SMS-delivered behaviour change interventions have been published and corroborate the findings of our earlier review [34-38].

### Step 3 Pre testing

In this development phase the feasibility and acceptability of the proposed SMS content, structure and frequency was tested in a qualitative study design. Participants were recruited through a local Playgroup and were eligible if they had a child aged under 5 years, owned a mobile telephone, were currently not meeting physical activity guidelines, and had intentions to increase their physical activity in the next 30 days. Participants provided written, informed consent and then participated in a F2F interview to collect details relevant for tailoring SMS content (i.e., their name, child’s name and physical activity preferences). During the two week test period participants were sent five MobileMums SMS per week at random times during the day between 8 am and 6 pm. SMS were generated using manual tailoring processes and sent using an off-the-shelf software package from a local telecommunication provider. This package used Microsoft Outlook as the user interface and did not allow for bulk sending of SMS to participants nor automated processing of incoming SMS from participants. After two weeks, a semi-structured F2F feedback interview was conducted, which included questions about preferred SMS frequency, timing, language and content. To explore language-to-text translations, participants were asked to convert a verbalised physical activity message to an SMS they would send to a friend. Participants were then asked to show the interviewer their phone so that the SMS could be recorded from the screen. The feedback interviews were audio recorded, transcribed verbatim and thematically analysed. A University Human Research Ethics Committee approved the study design.

Twelve participants aged from 17 to 39 years participated in the pre-testing. All women who consented to participate completed the pre-testing. One quarter (3/12) of participants had a weekly household income below AUD $600 and for one quarter (3/12) Year 10 was their highest level of education.

#### *SMS frequency and timing*

Participants recalled receiving between 4 and 11 SMS over the two week period (10 were sent), and most found the SMS frequency was acceptable. One participant noted that although the SMS were *“probably a little bit excessive, it was still good because it doesn’t hurt to get a text”.* Another participant who reported the SMS to be too frequent explained that she only thought this when a *“SMS was sent late afternoon one day followed by early in the morning the next day”* (i.e., based on the break between receiving SMS rather than the frequency).

#### *Treatment of SMS*

Participants reported reading all of the SMS sent to them. One participant indicated that occasionally she did not read the SMS straight away but waited until a time that suited her. Most participants stored the SMS after reading them and discussed storing SMS so they could refer to the details at a later stage (particularly SMS with details of local physical activity opportunities).

#### *SMS language*

All participants reported the SMS were easy to read and understand. When participants were instructed to write an SMS to a friend, the resulting SMS language and content varied (see Table 1). Participants used conversational language and tone, and used very little punctuation in the SMS they wrote. Participants also used a wide range of abbreviations, but common words across participants were always abbreviated such as: you = u; to = 2; for = 4; today = 2 day; and, Tuesday = tues. These common abbreviations were used hereafter in MobileMums SMS (see Table 1).

**Table 1:** Examples of participant’s language-to-text translation when instructed to type a SMS to a friend about a MobileMums-related topic

**Verbal instructions to write SMS**	**Exact SMS that participant typed on her phone**
· Get your friend to come to a walking group with you. The walking group is at Apex Park on Tuesday at 9 am. Let them know there is childcare too.	· Walking group @ Apex 9 am tues childcare for free
	· Do you want 2 join a walking group at apex park on tues at 9 am
	· Hey darl can u cum ta walking thing on Tues at 9 starts at apex
· Tell your friend that exercise can help her lose weight and reduce her stress levels.	· Hi Julie, exercise helps you lose weight and reduce stress. Wendy
	· Jen u cn lose fat n stress less if u exercs
	· Kerry if u exerc u dont get fat and stress
· Get your friend to ask her partner to look after the kids so you can go for a walk together.	· Hi Jill, ask Greg to look after the kids so we can go for a walk. Sally
	· Go 4 walk 2 day? get gav 2 look after kids
	· Hey I was wondering if use arnt doing anything would brad mind the kids while we go for a walk and catch up.

#### *SMS Content*

Most participants thought the SMS content was acceptable, suggesting no changes. One participant suggested that it would be good to let the mums know that other mums were receiving these messages and would be attending the local physical activity opportunities that were promoted. Most participants stated that the SMS content was not new to them but that it prompted them to exercise. SMS that presented unknown information to participants included those that: promoted new opportunities for exercise in the local area; informed of the relationship between exercise and breastfeeding; and, reminded them that incidental or household activity can be classified as exercise, if completed at appropriate intensity and durations. Participants liked the SMS that taught them new things, but also liked being reminded about content they already knew. It was also noted that women used the term ‘exercise’ (rather than ‘physical activity’) when discussing the content of the SMS and typing SMS to their friend. Our previous qualitative research has shown that women with young children perceive the term ‘exercise’ to represent moderate-to-vigorous intensity physical activity [39], which is what MobileMums aimed to promote.

The key findings from this development step, which informed the next iteration of MobileMums, were that: the maximum proposed frequency of 5 SMS/week was acceptable; the SMS language was acceptable; some abbreviations (e.g., u, 2, 4) were commonly used; the term ‘exercise’ should be used in MobileMums content; SMS that include details of promoted local exercise opportunities were likely to be stored by women for later reference; and, some women may want to connect with other mums who were also receiving the program.

### Step 4 pilot RCT

In line with the goals of Step 4 [12], the first complete version of MobileMums was evaluated in a pilot randomised controlled trial (RCT) compared to a minimal contact control group [40]. This first iteration of MobileMums was initiated with an individual, F2F counselling session with a trained behavioural counsellor in a community facility. The aim of this F2F session was to build rapport and a sense of accountability with the MobileMums counsellor, establish behavioural skills (e.g., goal setting, self monitoring), and collect information in order to tailor the SMS. Participants received tailored SMS for 12 weeks (frequency 2–4 SMS/week). In addition to these SMS, participants also received a weekly goal check SMS that asked them to reply and indicate whether they had reached their exercise goal or not (i.e., participants received a maximum of 5 SMS/week). Based on the participant’s reply to this goal check SMS, they received a tailored SMS response. Participant’s also received a telephone follow-up call with the behavioural counsellor six weeks into the program, and support from a self-nominated MobileMums support person (who also received two tailored SMS each week). Throughout the program participants had access to: information brochures on physical activity; a A5 sized refrigerator magnet designed to facilitate goal setting and self-monitoring; and an online forum within a community-based website to assist women to connect with one another.

Eighty-eight women (mean age 30 years ±SD 6 years) participated in the pilot RCT, which involved outcome assessments at baseline, 6-weeks (mid-intervention) and 13-weeks (end of intervention). For a detailed description on this intervention evaluation see Fjeldsoe, Miller, & Marshall (2010). Briefly, the results showed that MobileMums produced short-term increases in the frequency of self-reported moderate-to-vigorous intensity physical activity, in the range of 1.32-1.82 days/week [40]. We also found that short-term (baseline to 6-weeks) changes in goal setting skills and self efficacy significantly mediated the short-term impact of MobileMums on moderate-to-vigorous intensity physical activity [41]. Process evaluation with 34 women who received the MobileMums program indicated that the women regularly replied to weekly goal check SMS and frequently used the refrigerator magnet (about two thirds (22/34) were using the magnet daily post intervention). Almost all (33/34) of the intervention participants reported reading all of the MobileMums SMS they received. However, the on-line MobileMums discussion forum was not used at all.

Half of the intervention participants (17/34) rated MobileMums as ‘extremely useful’ or ‘useful’ and a further 14 participants rated it as ‘somewhat useful’ in supporting their exercise goals [40]. The behavioural counsellor noted that a major barrier to intervention engagement for participants was the location of the initial F2F session in a community facility.

Post-intervention interviews conducted with 11 women confirmed that program satisfaction was high [40]. Participants particularly liked: that MobileMums focused on their needs (and not their children’s needs); getting information about local exercise opportunities (although they wanted more details on costs, childcare etc.); the refrigerator magnet; and the weekly goal check SMS. These women thought that their self-nominated MobileMums support person was not useful and attributed this to competing demands for the support person’s time and attention (i.e., full-time employment).

Outcomes from the pilot RCT and follow-up interviews that informed further refinement of the MobileMums program included: 1) women needed greater flexibility regarding the location of the initial F2F consultation with the behavioural counsellor (i.e., their home rather than a location determined by the counsellor); 2) the role of the support person required clearer definition to ensure women selected the most appropriate person in their life and the support person SMS needed to more directive; 3) the on-line discussion forum was not used so another option for women to connect with one another should be identified; and, 4) access to additional information (beyond that offered in the SMS) regarding the promoted local exercise opportunities was desirable.

### Step 5 qualitative research for intervention refinement

Prior to developing the next iteration of MobileMums, we convened a steering group of health professionals and service delivery agents who were interested in widespread dissemination of a physical activity intervention for women with young children (i.e., maternal health nurses from the public hospital, child health nurses from community organisations, local government health promotion officers). The steering group reviewed the findings from the pilot RCT and advised that two key facilitators for the dissemination of MobileMums into practice would be: 1) an automated software program to generate and send SMS and manage incoming SMS replies from participants, to minimise the need for personnel time, and 2) a formalised handbook to guide the systematic delivery of the F2F consultation, since in practice the program would be delivered by multiple staff (not a single behavioural counsellor as in our pilot study).

After attracting additional research funding we were able to engage a professional software developer to produce a MobileMums-specific web-based application to send and receive SMS. This application allows administrators (i.e., MobileMums behavioural counsellors) to enter participant data into a web-based interface, which is then stored in a secure database (supported by *mySQL*). The program then merges these participant fields with pre-developed frameworks to generate personalised SMS and sends the SMS according to the pre-determined schedule (i.e., frequency and days/week). Each SMS is sent within a three-hour window (between 6 am and 7 pm) based on the participant’s preference. Most importantly, the software uses algorithms to automatically reply with a tailored, behaviourally-appropriate response to the participant’s replies to the weekly goal check SMS (yes/no reply requested). If participant’s goal check SMS response does not match the predicted reply words (i.e., less than 5 characters and starting with “y” or “n”), the administrator is notified via email so that the SMS can be read and an appropriate SMS response triggered. Based on findings in Step 4, we also created a dedicated MobileMums website that included detailed physical activity information, a discussion forum with improved usability (e.g., thread tracking) and a searchable exercise directory of local opportunities.

We created a comprehensive MobileMums Training Guide to guide the content of the initial F2F and 6-week counselling sessions and train behavioural counsellors. This book contains reviews of the benefits of exercise in the postnatal period, and the theoretical underpinnings of MobileMums. It also contains information on effective counselling techniques to support behaviour change. Finally, the training guide describes a 10-step process to guide the initial F2F counselling session and a 6-step process to guide the 6-week follow-up telephone counselling call. We also created another book, the MobileMums Participant’s Handbook, to outline the steps in each of the counselling sessions and provide participants with space to record decisions made during their behavioural counselling sessions.

Six women with young children were recruited from the sample of participants who had been involved in the MobileMums pilot RCT (described in Step 4) to test the initial F2F consultation with the behavioural counsellor (at a preferred location) and program initiation procedures for support person registration. Women were also asked to visit the new MobileMums website (specifically the discussion forum and on-line exercise directory) and to provide written feedback on these web-based components via a mailed questionnaire. Four of the six women received the first two weeks of the automated MobileMums SMS: this allowed us to test the software’s functionality in terms of automated responses to participant’s goal check replies. Following this, the women completed a semi-structured telephone interview to give feedback on the F2F consultation, SMS content, program materials (e.g., on-line exercise directory), and support person registration process.

The feedback interviews provided further ideas for refining the program. These were: the need to include more affordable exercise opportunities in the on-line exercise directory, in particular walking routes; a desire to have a description of the exercise directory entries from the perspective of a woman who had engaged in the activity; and, a preference for a Facebook^©^ group over the MobileMums discussion forum. Through the software function testing we identified the need to screen participant replies if more than four characters were included in the reply (i.e., more than a “yes” or “no” response was given). This refinement was necessary since some participants provided extra information if for example, they didn’t meet their goal (e.g., *No, but only because I was sick*). In these instances the automated reply sometimes seemed insensitive or irrelevant and undermined the developing virtual relationship. Further to this we developed a protocol to guide when it was ethically important to personalise the SMS reply. This included if the participant goal check reply referred to: personal or child health issues; domestic violence; relationship difficulties; depression, grief, or crisis management; financial difficulties; as well as other issues directly related to their MobileMums participation. This protocol was added to the MobileMums Training Guide.

A final round of user testing was conducted with three women and their support people. The complete 12-week SMS program was administered to identify potential bugs within the web-based application and automatic response mechanisms. This round of user testing identified possible risk of habituation to receiving the MobileMums SMS at exactly the same time each day (e.g., precisely 9:00 am). Therefore, the program was modified to ensure each SMS was sent at a randomly selected time within the three hour window. Also, the women commented that the SMS they received in response to their goal check replies were too immediate (within seconds), revealing the automated nature of the program and posing a threat to the earlier-identified need for rapport between the SMS recipient and sender (see Step 2). In response to this, the web-based application was modified so that the goal check replies are sent between 5- and 60-minutes after the participant response was received.

### Final version of the MobileMums intervention

This section describes the MobileMums program as it currently stands being evaluated within a community-based RCT. Each component of the MobileMums intervention is described separately and in more detail below. As an overview, the 12-week intervention is initiated with a semi-structured F2F consultation with a trained behavioural counsellor, after which participants receive five tailored SMS/week during Weeks 1 to 4 and four SMS/week during Weeks 5 to 12. During Week 6 participants receive a follow-up telephone counselling call (TC) from their behavioural counsellor. Throughout the intervention, participants have ongoing access to their MobileMums Participant Handbook, MobileMums website with on-line exercise directory, MobileMums Facebook^©^ group, MobileMums refrigerator magnet, and information brochures. During the F2F consultation each participant is asked to identify a MobileMums support person. The support person also receives tailored SMS encouraging them to offer instrumental, emotional, or informational support to assist the participant to be more active.

In finalising the content this version of the MobileMums intervention, we revisited how we operationalised the constructs of the Social Cognitive Theory (self efficacy, goal setting skills, outcome expectancies, social support and perceived environmental opportunity) into behaviour change techniques [3]; (see Additional file 1). Particular care was taken to ensure that within each intervention component (i.e., SMS, initial F2F consultation) there was even coverage of all five of the theoretical constructs (e.g., equal emphasis on each construct across the 52 SMS sent to each participant). In designing the intervention content, certain theoretical constructs (i.e., outcome expectancy, perceived environmental opportunity) were targeted more often in the early stages in the intervention (i.e., first 6 weeks) than later in the intervention. This differential timing of targeting constructs was based on evidence that outcome expectancies are particularly important in the early adoption phase of physical activity behaviour change [14,42] and was confirmed by the findings of Steps 3 and 4 that women wanted to know what exercise opportunities existed in their local areas to assist them to start new routines.

*Initial F2F consultation:* the aim of this consultation is to: establish rapport between the participant and the counsellor; to gather information in order to tailor the SMS content; and, to initiate the behaviour change process. The behavioural counsellor guides the participant through a 10-step process outlined in the MobileMums Participant Handbook, which participants can make notes in and keep. The 10-step process guides participants to: 1) establish the purpose and scope of the session; 2) review their current exercise patterns based on baseline assessment data; 3) discuss realistic outcome expectancies of regular exercise; 4) negotiate required social support for regular exercise and identify a support person; 5) identify potential cues and environmental opportunities for regular exercise; 6) set a SMART exercise goal; 7) set weekly rewards for goal attainment; 8) plan a weekly schedule for exercise; 9) identify potential barriers and feasible solutions; and, 10) evaluate their commitment to the plan. During this consultation, participants also receive state-of-the-art information brochures on exercise and a MobileMums refrigerator magnet. Participants are also informed of how to access the MobileMums Facebook^©^ group, MobileMums website, and online exercise directory. This consultation occurs in a location chosen by the participant (e.g., their home) and lasts between 25- and 45-minutes.

*Behavioural counsellor:* the personnel employed to deliver the initial F2F and 6-week consultations were required to hold a health-related bachelor degree and government clearance for working around children. Counsellors underwent extensive training in the principles of promoting moderate-to-vigorous intensity physical activity, the constructs of the Social Cognitive Theory, effective counselling skills (e.g., active listening), and familiarisation with issues specific to the target group. The counsellors were provided with the MobileMums Training Guide, had multiple one-on-one training sessions and engaged in role play prior to contact with participants.

**Figure 2:**
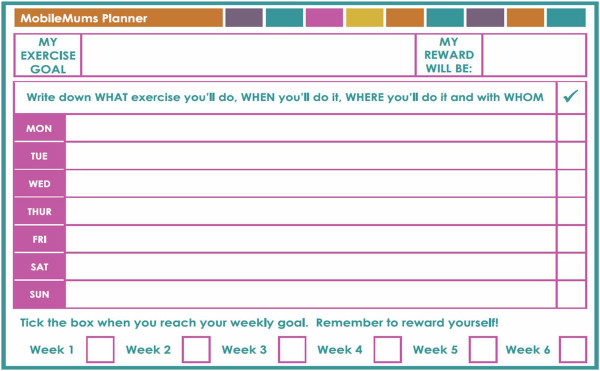
MobileMums refrigerator magnet.

*MobileMums refrigerator magnet:* the refrigerator magnet is 15 × 21 cm and includes a magnetic erasable pen (see Figure 2). The magnet has a daily planner for participants to plan and track their exercise across the week. During the F2F consultation, the behavioural counsellor guides the participant to write on their magnet their exercise goal, weekly reward, and exercise plan (‘What, When, Where and with Whom’ they will exercise) for the first week of the program. Participants are encouraged to update the reward and plan for each subsequent week of the program and SMS prompt participants to use the magnet regularly. The support person SMS also refer to the magnet to encourage support people to facilitate the planned sessions of exercise, to monitor participant’s weekly goal progress and to assist in operationalizing rewards.

*Facebook*^*©*^*group:* participants are informed of the MobileMums Facebook^©^ group (a private page), which they could request entry to via sending a friend request to the MobileMums coordinator. The coordinator posted an initial comment on the ‘wall’ of the Facebook^©^ group, which welcomes women and encourages them to introduce themselves to other mums in the group. Communications on the Facebook^*©*^ page are monitored weekly by the coordinator for incorrect or offensive information, but is otherwise not interfered with. Women can also send private one-to-one messages to other women in the group.

*MobileMums website and on-line exercise directory:* the website contains information on evidence supporting the benefits of postnatal exercise, a link to the Facebook^©^ group, and photos and testimonials of previous MobileMums. The website also includes the exercise directory, which allows participants access to a searchable directory of opportunities to exercise in their local area. These opportunities are categorised by type (i.e., Walking Routes, Parks, Group Classes, Gyms, Personal Training, Swimming & Aqua Aerobics) and are searchable by map. Each entry in the directory includes details such as: location, contact details, facilities available (including on-site childcare), associated costs, potential risks, and a description written by the MobileMums behavioural counsellor who reviewed the activity. Participants also receive a hard-copy of the directory specific to their local neighbourhood during the initial F2F consultation.

*MobileMums SMS:* the SMS content is tailored to the women’s exercise goal, their rewards and expected outcomes for reaching their goal and the neighbourhood that they live in (see Additional file 1 for example SMS). In addition, the SMS wording is tailored based on the: participant’s name and gender; support person’s name and gender; youngest child’s name; and name of participant’s behavioural counsellor. Women receive five SMS per week for four weeks (1 goal check SMS and 4 others) and four SMS per week for the remaining eight weeks (1 goal check SMS and 3 others). The goal check SMS is sent every Monday and asks participants whether or not they met their weekly exercise goal. If the participant replies to the goal check SMS then they receive a tailored SMS in reply (i.e., an additional SMS to the 4 or 5 per week). This goal check reply is tailored to whether the participant reached their goal or not (e.g., *Jenny, its OK. It might take time 2 get in2 the swing of things. Think about what went wrong & find at least 1 way 2 make it easier this wk. Jacqui -MobileMums*).

*MobileMums support person SMS:* during the initial F2F consultation each participant is guided to identify the tasks that would be supportive to them reaching their exercise goal and then identify the most appropriate person in their lives to fulfil these tasks (e.g., partner, family member, friend, or neighbour). Participants are then given a letter inviting their identified person to be their MobileMums support person for the duration of the 12-week program. The letter asks the potential support person to log-in to the MobileMums website to provide consent, some personal details (i.e., exercise habits, relationship to participant) and their mobile telephone number. Once they have entered this information on-line an automated SMS is sent to them requesting that they reply to confirm their consent. Consenting support people receive three personalised SMS per week within their three hour nominated send time (e.g., 9–12 am). These SMS provide prompts and ideas to provide instrumental, emotional, or informational support to assist their participant to reach her exercise goal. Every second week one of the three SMS that the support person receives is tailored to whether the participant they support responded to their weekly goal check (e.g., *Luke, congratulate Jenny. She met her goal last wk. Can u help make time 4 her reward? Its a bubble bath. Jacqui- MobileMums*).

*6-week telephone consultation:* during Week 6 each participant receives a follow-up telephone consultation with their behavioural counsellor. This consultation is guided by a 6-step process outlined in the MobileMums Participant Handbook and aims to reflect on participant progress during the first six weeks and to update exercise goals, rewards and plans for the subsequent six week period. Data collected during this call are entered into the online database by the behavioural counsellor, so that SMS sent during Weeks 7 to 12 are relevant to the participant’s most recent exercise goal and rewards.

Copies of any MobileMums resources are available from the last author on request.

## Discussion

This paper described the process of developing the MobileMums physical activity intervention and the final version currently undergoing efficacy testing. This level of detail is rarely provided in research literature, but is essential if the field of behaviour change interventions is to advance. There are currently special interest groups collaborating with peer-reviewed journals to introduce protocols that require authors to include this level of detail in published intervention evaluations [2,43]. Currently, most journals require authors to comply with CONSORT guidelines when reporting on behavioural interventions, which includes a single check point about ‘precise details of the interventions intended for each group and how and when they were actually administered’ [44]. Generally however, this is covered by minimal details of what and when. If further details of where, how and why are to be included in intervention descriptions then the length and formatting restrictions of some journals will need adjusting to accommodate the additional information.

An issue experienced in the development of the MobileMums intervention which may be relevant for other mHealth interventions, is the time required to develop the program, secure funding, then systematically develop and evaluate the program. This delay in implementation and evaluation means that by the time results are published and practice is impacted, the technology has often already progressed. Step 1 of the MobileMums development process commenced in 2006. During our initial focus groups women felt that the use of smart phone applications was not feasible due to the minority of women who had access to this technology. Six years later, the high penetration of smart phones may have changed this perception. There is a growing body of published behaviour change interventions delivered via smart phone applications [45,46]. However, some of the key facilitators of the success of MobileMums have been unique features of SMS and may not transfer to applications. For example, women expressed perceived accountability to the behavioural counsellor because the two-way SMS communication maintained a ‘human aspect’ to the intervention. Throughout the development process the MobileMums intervention content remained grounded in Social Cognitive Theory. While it has been noted that most traditional health behaviour change theories are static in nature and may not provide adequate guidance to mHealth interventions [38], the Social Cognitive Theory served MobileMums well enough to guide the behaviour change techniques. However, in future iterations of MobileMums it may be possible to adapt the content of the SMS based on the pattern of previous weekly goal check replies (rather than just the reply to each weekly goal check) or on objectively assessed physical activity patterns detected by the mobile handset either via Bluetooth connection to a wrist-worn accelerometer [47] or via accelerometers that are now commonly incorporated into the handset [48]. These advances in technological capability may require researchers to embrace more dynamic theories and to follow more complex development processes, such as those outlined in the SMART framework [11].

Throughout the development process the MobileMums intervention has evolved to include other non m-health delivered components (e.g., participant handbook, Facebook group), however the majority of intervention contact with participants and their support person is via SMS. As described in this paper the necessary additions of other delivery methods have been based on the findings of the comprehensive formative development process undertaken with the target group. Future research should explore how the non m-health components of the intervention could be translated to sole mHealth delivery, and how the effectiveness of the program is affected as a result.

## Conclusions

The process of developing MobileMums involved many phases of formative research and pilot testing. The resultant semi-automatic program has evolved from its original manually managed iteration but has maintained strong theoretical-grounding throughout the development process. Results of a current community-based randomised controlled trial to evaluate the final version of MobileMums described in this paper will inform further translation of the intervention beyond researcher administration into community-based practice (ACTRN12611000481976).

## Abbreviations

F2F: Face-to-face; MOST: Multiphase Optimisation Strategy; RCT: Randomized controlled trial; SMS: Short messaging service; SMART: Sequential Multiple Assignment Randomized Trial.

## Misc

Brianna S Fjeldsoe Yvette D Miller, Jasmine L O’Brien, and Alison L Marshall contributed equally to this work

## Competing interests

The authors declare that they have no competing interests.

## Authors' contributions

BF, YM and AM conceptualised the development of MobileMums. BF and JO collected the data used in the development of MobileMums. All authors were involved in analysing and interpreting the data. All authors read and approved the final manuscript.

## Supplementary Material

Additional file 1 **MobileMums intervention content mapped to behaviour change techniques (Michie et al., 2011) and Social Cognitive Theory.** A table displaying how each behaviour change technique (from the Michie et al., taxonomy) is targeted in the MobileMums intervention and how these relate to the theoretical basis of Social Cognitive Theory. Click here for file
