# Supplementary material for: Iterative development of MobileMums: a physical activity intervention for women with young children
Source: Int J Behav Nutr Phys Act. 2012 Dec 20;9:151. doi: 10.1186/1479-5868-9-151 (PMC3541201; doi:10.1186/1479-5868-9-151)
Supplement: Additional file 1 — MobileMums intervention content mapped to behaviour change techniques (Michie et al., 2011) and Social Cognitive Theory. A table displaying how each behaviour change technique (from the Michie et al., taxonomy) is targeted in the MobileMums intervention and how these relate to the theoretical basis of Social Cognitive Theory. [file 1479-5868-9-151-S1.doc]

**MobileMums intervention content mapped to behaviour change techniques (Michie et al., 2011)** and Social Cognitive Theory

| **Behaviour change technique  (listed by taxonomy number)** | **Theoretical construct** | **MobileMums intervention component** |
| --- | --- | --- |
| 2. Provide information on consequences of behaviour to the individual *(tailored to postnatal women)* | OE | **F2F:** discuss most common positive and negative expectations of increasing exercise and provide evidence-based information on realistic expectations  **SMS:** 8 targeting technique (e.g. *Jenny. Don’t feel guilty 4 taking time out 2 exercise, mums say they r more patient & understanding because they exercise. Jacqui -MobileMums*)  **Brochures:** provide summary of evidence for positive and negative outcomes of regular exercise in postnatal period  **Website:** provide summary of evidence for positive and negative outcomes of regular exercise in postnatal period |
| 4. Provide normative information about others’ behaviour | GSS, SS | **SMS:** 5 targeting technique (e.g. *Want 2 try a group exercise class or swimming? Check out what other mums are doing on the facebook group. Jacqui -MobileMums*)  **Facebook©:** women share exercise goal progress, strategies for planning exercise and overcoming barriers  **Website:** pictures and testimonials from previous MobileMums |
| 5. Goal setting (behaviour) | GSS, SE | **F2F:** negotiate initial exercise goal with behavioural counsellor  **TC:** discuss exercise goal progress and revision with behavioural counsellor  **SMS:** 6 targeting technique (e.g. *Its almost time 2 plan ur SMART goal 4 the next 6 wks of MobileMums. See p.9 of ur handbook 4 tips. Jacqui-MobileMums*)  **Magnet:** record weekly exercise goal |
| 7. Action planning | GSS, SE | **F2F:** discuss plan for first week of exercise (what, when, where, with whom?)  **Magnet:** plan and record daily sessions (what, when, where, with whom?)  **TC:** discuss plan for next week of exercise (what, when, where, with whom?)  **SMS:** 7 targeting technique (e.g. *Jenny. u told me you want 2 have more energy. 2 achieve this u need 2 stick 2 ur MobileMums plan. Check ur planner magnet 2day. Jacqui-MobileMums*) |
| 8. Barrier identification/ problem solving | GSS, SS | **F2F:** identify potential barriers and negotiate solutions  **TC:** discuss experienced barrier over past 6wks and negotiate solutions  **SMS:** 8 targeting technique (e.g. *Jenny, its OK 2 miss a day now & then, we all do. The trick is 2 get back in2 it ASAP. Review the strategies we planned in ur handbook. Jacqui-MobileMums*)  **Facebook©:** women share ideas for overcoming barriers |
| 10. Prompt review of behavioural goals | GSS, SE | **SMS:** 11 weekly goal check SMS (e.g. *Jenny did u do all ur planned exercise last wk? Check ur planner magnet & text me back yes or no. Jacqui-MobileMums*)  **Magnet:** planning daily sessions (what, when, where, with whom?) |
| 13. Provide rewards contingent on successful behaviour | GSS, SE | **F2F:** selection and recording of self-reward based on weekly goal attainment  **SMS:** 4 targeting technique (e.g. *Jenny, remember the reward u chose 4 reaching ur goal this wk was a bubble bath. U’ll deserve it if u work 4 it. Jacqui-MobileMums*) + 22 targeting technique if participant replied to weekly goal check (e.g. *Well done Jenny! Gr8 start. Make sure u find time 4 ur reward: a bubble bath! Jacqui-MobileMums*)  **Magnet:** record and track weekly rewards |
| 16. Prompt self-monitoring of behaviour | GSS | **SMS:** 3 targeting technique (e.g. *OK Jenny its a new wk so lets start fresh. Use ur planner magnet 2 remind u of ur scheduled sessions & stick 2 it. Jacqui-MobileMums*)  **Magnet:** track daily sessions, weekly goal attainment and rewards |
| 18. Prompting focus on past successes | SE, OE | **F2F:** discuss previous types of exercise, reasons for stopping  **TC:** reflect on first 6 weeks of exercise goal attainment  **SMS:** 6 targeting technique (e.g. *Jenny take a minute 2 think about how much better u feel after an exercise session. Remember this next time u don’t feel like doing it. Jacqui-MobileMums*) |
| 19. Provide feedback on performance | GSS, SE | **F2F:** reviewing objectively measured physical activity from baseline assessment  **SMS:** 22 targeting technique if participant replied to weekly goal check (e.g. *Fantastic! It must feel good 2 prove 2 urself u can do it – enjoy that feeling. Jacqui-MobileMums*) |
| 20. Provide information on where and when to perform the behaviour | PEO | **SMS:** 10 targeting technique including details of where, when, cost (e.g. *Hi Jenny. MobileMums r enjoying aqua aerobics at Redcliffe Aquatic Centre. Tues & Thurs 4pm. Costs $6.50. Childcare available. Jacqui-MobileMums*)  **Website:** searchable on-line exercise directory |
| 21. Provide instruction on how to perform the behaviour | SE | **F2F:** discuss specific steps required to reach weekly exercise goal (e.g. prepare clothing, organise childcare, call local gym facility)  **SMS:** 4 targeting technique (e.g. *Jenny 4 exercise 2 count it must b moderate intensity 4 at least 10mins each time. So u should b able 2 talk but not sing while exercising. Jacqui-MobileMums*) |
| 23. Teach to use prompts/cues | GSS, SE, | **F2F:** discuss positive and negative cues to exercise  **SMS:** 3 targeting technique (e.g*. Hi Jenny. Always have ur exercise clothes clean & ready. Don’t let it b an excuse. Leave ur shoes where u can c them 2 remind u. Jacqui-MobileMums*) |
| 24. Environmental restructuring | PEO | **F2F:** discuss specific steps to make home environment more conducive to exercise (e.g. put magnet on fridge as reminder, put exercise shoes at end of bed) |
| 29. Plan social support/ social change | SS | **F2F:** discuss ‘ideal’ of required support, then identify best person in participant’s life to fit this role, discuss techniques for negotiating required support  **SMS:** 9 targeting technique (e.g. *Jenny. Remember Luke wants 2 support u. Make sure he knows what ur MobileMums goal is & what he can do 2 help u meet it. Jacqui-MobileMums*)  **SMS to Support Person:** 36 targeting technique (e.g. *Luke. Remind Jenny 2 reward herself when she reaches her weekly exercise goal. Ask what u can help with this wk. Jacqui-MobileMums*)  **Facebook© :** women from same geographical area discuss goals, strategies and barriers, seek ‘walking buddies’ via private messaging within group |

***Note:*** SE: self efficacy, GSS: goal setting skills, OE: outcome expectancies, SS: social support, PEO: perceived environmental opportunity, F2F: initial face-to-face consultation, TC: telephone counselling session at 6 weeks, SMS: short messaging service
